# A Rare Adrenal Incidentaloma That Mimics Adrenocortical Carcinoma

**DOI:** 10.1155/2018/9607972

**Published:** 2018-06-07

**Authors:** Kedar Lavingia, Ramyar Torabi, Samuel W. Kim, Marybeth S. Hughes, Eric C. Feliberti, Roger R. Perry

**Affiliations:** Division of Surgical Oncology, Department of Surgery, Eastern Virginia Medical School, 825 Fairfax Ave, Norfolk VA 23507, USA

## Abstract

**Objective:**

We present a case of an adrenal hemangioma, an uncommon cause of an adrenal mass, and review the clinical presentation, work-up, and management of adrenal incidentalomas.

**Background:**

A 64-year-old male was found to have a right adrenal incidentaloma during work-up for elevated liver transaminase levels, later found to be from hepatitis C. The mass was suspicious for adrenocortical carcinoma on CT imaging. Biochemical evaluation revealed no evidence of function. He underwent an open right adrenalectomy. The mass was found to be an adrenal hemangioma on histopathologic analysis.

**Methods:**

This is a case report with pertinent review of the diagnosis and management of adrenal incidentalomas.

**Results:**

Adrenal hemangiomas are rare, benign, nonfunctional tumors typically found during imaging for other reasons. As illustrated by this case, they appear similar to adrenocortical carcinoma on CT imaging. The diagnosis is usually not made prior to surgical resection.

**Conclusion:**

Adrenal hemangioma is a rare nonfunctional adrenal incidentaloma that displays atypical features on CT imaging. The suspicion for adrenocortical carcinoma usually prompts adrenalectomy.

## 1. Introduction

Adrenal hemangiomas are rare benign nonfunctional tumors typically found incidentally with imaging. Since the first reported case in 1955, there have been about 90 reported cases in the literature [1]. Most adrenal hemangiomas are cavernous, while a rare capillary type has also been seen [2]. Patients are usually asymptomatic, though the lesion carries a small risk of spontaneous hemorrhage. Adrenal hemangiomas are radiographically difficult to distinguish from adrenocortical carcinoma and usually undergo surgical resection for this reason.

## 2. Case Presentation

**Figure 1:**
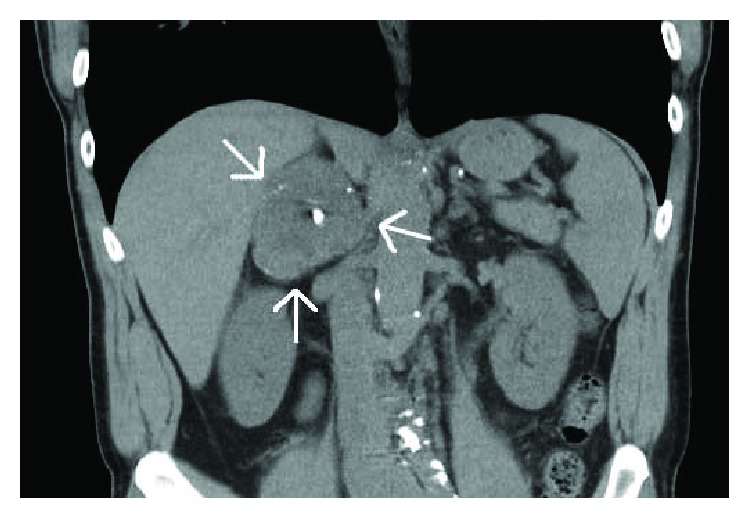
Adrenal-protocol CT abdomen, coronal demonstrates a 5 × 6.7 × 7 cm right adrenal mass (white arrows) with areas of calcification. Hounsfield units (HU): nonenhanced 36 HU (>10 HU indeterminate for adenoma), enhanced 84 HU, and delayed 73 HU. Absolute washout 22.9% (<60% indeterminate for adenoma) [3].

A 64-year-old white male with no prior medical history presented to his primary care physician for routine follow-up. There was no history of hypertension. During work-up for elevated liver transaminases, he was found to have hepatitis C. Before initiation of Harvoni, he underwent CT imaging of the abdomen with contrast which found a 5 × 6.7 × 7 cm right adrenal mass (Figure 1). On physical examination, he was afebrile with a pulse of 47 and normotensive at 118/68. His abdominal exam was nontender, nondistended, without masses, or hernias. Review of systems was negative for abdominal pain, hypertension, weakness, palpitations, headache, diaphoresis, or weight gain. He was a current smoker with a 33 pack-year history. He had no history of endocrine disease. His family history was significant only for a father with pancreatic cancer. His remaining laboratory values were within normal values including a normal potassium value. The patient was seen by the endocrine service for evaluation, and biochemical work-up revealed that the ACTH level was 9.1 pg/ml (nl 7.2–63.3); AM cortisol was normal at 10.01 mcg/dl, and 24-hour urine metanephrines was less than 50 mcg (nl).

On CT imaging, the right adrenal mass contained scattered calcifications with small regions of fat. It enhanced in a peripheral globular fashion with central progression. The absolute contrast washout of 22.9% was indeterminate for adrenal adenoma (Figure 1). The mass was noted to abut but did not appear to invade the adjacent liver, right kidney, and inferior vena cava. There was no adenopathy or free fluid. There was no evidence of metastatic disease.

**Figure 2:**
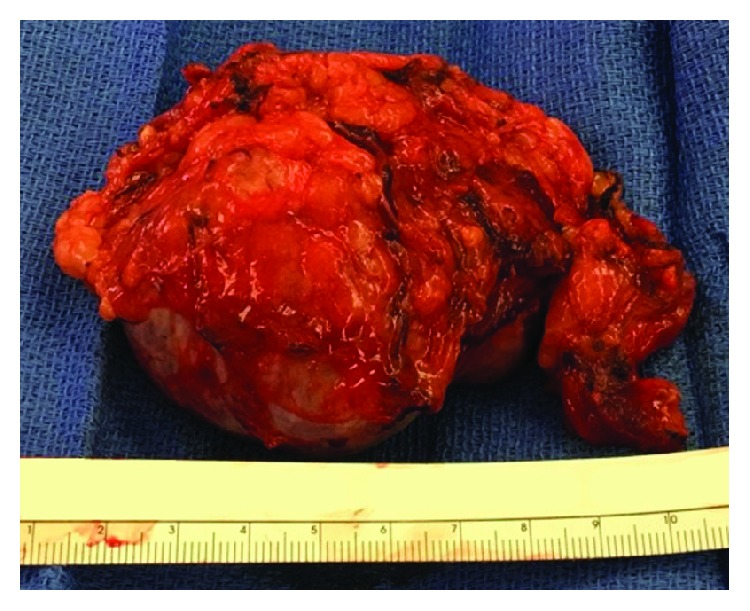
Resected specimen at the time of surgery. Histologic examination of the specimen revealed that the large dominant nodule was a benign cavernous adrenal hemangioma with hemorrhage (Figure 3).

**Figure 3:**
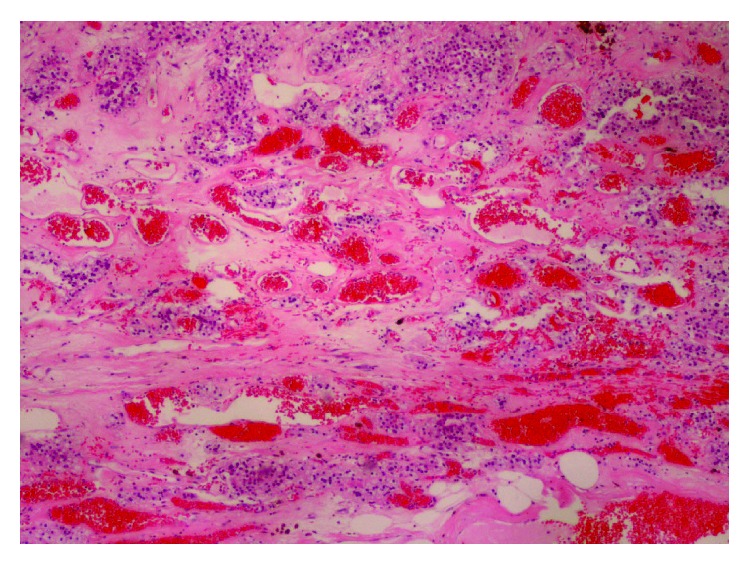
Histological examination with hematoxylin and eosin staining revealed multiple blood-filled spaces lined by an individual layer of vascular endothelium, typical of a cavernous hemangioma.

Due to the size and atypical features of the mass, right adrenalectomy was performed. An open thoracoabdominal approach was chosen due to the patient's low lying costal margin, the size of the mass, and retrocaval extension of the mass medially towards the vertebral body. The patient recovered well postoperatively and was discharged four days after surgery. The resected specimen weighed 126 grams and measured 7.5 × 6.5 × 4.7 cm on gross pathology (Figure 2). Within the specimen was a 6.4 × 5.5 × 4.7 cm intraparenchymal nodule, which on histologic examination proved to be a cavernous hemagioma (Figure 3). The patient has had no evidence of recurrence for nearly 18 months.

## 3. Discussion

An adrenal incidentaloma is an adrenal mass that is generally 1 cm or more in diameter found incidentally during abdominal imaging done for other reasons [4, 5]. In a review of 25 studies looking at a total of 87,065 autopsies, adrenal adenomas were found at a rate of 6%, with a range of 1–25% among the studies [4]. In a study of 520 subjects undergoing CT of the chest for lung cancer screening, 4.4% were found to have adrenal masses [6]. The prevalence of adrenocortical carcinomas among adrenal incidentalomas ranges from 1.2 to 12% among different studies [5]. The probability of finding an adrenal lesion increases with age and is approximately 0.2% between the ages of 20–29, while it is almost 7% in patients above 70 years old [4].

In a review of 2005 incidentally discovered adrenal masses, 82.5% were nonfunctioning benign lesions, 11.4% were functioning adenomas, and 7.2% were malignant lesions. Of nonfunctioning benign lesions, 61% were adenoma, 10% myelolipoma, 6% adrenal cyst, and 5.6% ganglioneuroma [7]. Of functional masses, 46.5% caused Cushing's syndrome, 44.7% were pheochromocytoma, and 8.8% were aldosteronoma. Of the malignant lesions, 65.3% were adrenocortical carcinoma and 34.7% were metastasis [7, 8]. Patients with adrenocortical carcinoma are usually 40–50 years old and have a poor prognosis.

The work-up of an adrenal incidentaloma includes, first, biochemical evaluation to determine if the mass is functional and, second, imaging to assess whether the mass appears malignant or benign. A careful history and physical examination may reveal specific signs and symptoms suggestive of a functional tumor. However, an asymptomatic patient should still be considered for biochemical testing, as 5% of tumors will demonstrate subclinical function. Of note, a pheochromocytoma must be excluded prior to any invasive procedure or surgery, since an untreated pheochromocytoma can lead to potentially lethal intraoperative hypertension.

A limited work-up for function was felt sufficient by the endocrine service. An occult pheochromocytoma was excluded [7]. The normal ACTH and AM cortisol levels were felt sufficient to exclude a functioning cortisol-secreting adenoma. Given the low normal ACTH level (<10 pg/ml), the possibility of low-level cortisol hypersecretion was not entirely excluded. An overnight 1 mg dexamethasone suppression test would have been helpful in this regard [7]. However, the results of this test would not have changed management. Subsequent guidelines have been published supporting routine use of the overnight 1 mg dexamethasone suppression test [9], which although more sensitive is less specific [3]. Biochemical evaluation to exclude aldosteronoma would have been of low yield based on lack of hypertension, normal serum potassium levels, and the CT size and appearance of the lesion [3, 10]. Similarly, in the absence of clinical manifestations, routine screening for sex hormone-secreting adrenal tumors is not recommended [4]^.^

In addition to testing the mass for function, the mass must be evaluated for the possibility of malignancy. Metastatic disease is frequently bilateral to the adrenal glands, and usually the primary tumor has previously been identified [4]. The two most important predictive factors of adrenocortical carcinoma are the size of the lesion and appearance on imaging [4]. Adrenocortical carcinomas are usually large (greater than 4 cm in diameter), irregular with unclear margins, heterogeneous with mixed densities, usually solitary and unilateral, vascular on contrast enhanced CT, and have delayed washout of contrast. Necrosis, hemorrhage, and calcifications are commonly seen in these lesions, which are often observed to grow rapidly (greater than 2 cm per year) [4]. In patients with no history of malignancy and a >4 cm adrenal mass which is indeterminate on imaging (as in our patient), there is consensus that surgical resection should be considered [3, 11]. FDG-PET/CT may be appropriate as part of preoperative staging [11]. In patients with known malignancy, FDG-PET/CT may be helpful in distinguishing benign adrenal masses from metastases [3, 11]. For a detailed discussion on adrenal imaging, one is referred to a recent review [3] and the American College of Radiology appropriateness criteria [11].

The mass in our patient had many of the imaging characteristics of adrenocortical carcinoma including size more than 4 cm, heterogeneous appearance, irregular enhancement pattern, delayed washout of contrast, and the presence of calcifications. Although calcifications are frequently present in adrenocortical carcinoma, calcifications are a nonspecific finding and may be seen in a variety of malignant and benign (hemorrhage, tuberculosis, and neuroblastoma) adrenal lesions [12], including hemangioma [1, 12]. The CT findings in adrenal hemangioma overlap with those of adrenocortical carcinoma and other lesions [1, 12–14]. Therefore, the diagnosis is usually not made prior to surgery [12, 14], as in our patient.

Of benign adrenal masses, adrenal hemangiomas are rare. While there are limited data, adrenal hemangiomas have been found in a 2 : 1 female to male ratio, are usually unilateral, and have been found most often in the 6th and 7th decades of life [2]. These lesions range from 2 to 25 cm in diameter and typically are larger than 10 cm [1]. One case report describes a 42 cm adrenal hemangioma [1]. While usually asymptomatic, patients with adrenal hemangiomas may present with abdominal pain due to mass effect or hypovolemic shock due to hemorrhage from the mass.

On histopathological evaluation, most adrenal hemangiomas are found in the adrenal cortex and consist of cavernous blood-filled sinusoidal channels that have eroded and displaced normal tissues [2]. These dilated vascular channels are lined by a single layer of vascular endothelium surrounded by a wall of collagen [12]. They are thought to be congenital lesions that enlarge over time because of vascular ectasia [12]. Less commonly, adrenal hemangiomas are capillary and include a small tuft of submucosal capillaries arranged in radiating loops or lobules. In both subtypes, there is variable hemorrhage, necrosis, degeneration, calcification, and fibrosis throughout the mass [2, 12].

Functional adrenal lesions, suspicious lesions, indeterminate lesions larger than 4 cm, or lesions that enlarge by 1 cm or more during observation should be considered for resection in medically fit patients [3, 4]. If functional studies are all normal and the adrenal mass is small (less than 4 cm) and does not appear malignant on imaging, the mass may be observed with repeat imaging for the first 6–12 months and then annually for 4 years [4]. In our case, the size and atypical characteristics on imaging lead to resection of the mass.

The surgical approach depends on the characteristics of the lesion. Most incidentalomas can be resected laparoscopically; however, an open surgical approach should be used if there is malignant potential or if the mass is large (greater than 6 to 10 cm) and appears difficult to remove [7].

## 4. Conclusions

Adrenal hemangioma is an uncommon, benign, nonfunctional tumor that should be considered during the work-up of an adrenal incidentaloma. Due to the difficulty in distinguishing the lesion from an adrenocortical carcinoma on imaging and due to the small risk of spontaneous hemorrhage, surgical excision is usually recommended. The diagnosis is usually not made prior to surgery. Outcome after resection is excellent and recurrence should be uncommon.
